# Adversarial Sequence Mutations in AlphaFold and ESMFold Reveal Nonphysical Structural Invariance, Confidence Failures, and Concerns for Protein Design

**DOI:** 10.34133/csbj.0142

**Published:** 2026-06-29

**Authors:** Jonathan Feldman, Maximilian Brogi, Jeffrey Skolnick

**Affiliations:** ^1^ College of Computing, Georgia Institute of Technology, Atlanta, GA, USA.; ^2^ Center for the Study of Systems Biology, Georgia Institute of Technology, Atlanta, GA, USA.; ^3^ School of Biological Sciences, Georgia Institute of Technology, Atlanta, GA, USA.

## Abstract

AlphaFold has transformed structural biology and spawned an ecosystem of derivative tools for protein design, binding prediction, and drug discovery. However, whether AlphaFold has learned generalizable biophysical principles as opposed to template-based pattern matching remains unclear—a distinction critical for applications beyond its training context. Here, we perform a systematic adversarial evaluation of AlphaFold 3 using point and deletion mutations across 200 proteins. Remarkably, predicted structures remain invariant to mutations of up to 40% of residues—including deliberately destabilizing substitutions—and to deletions of 10%. Notably, this invariance holds even for experimentally validated fold-switching proteins that are known to adopt alternative conformations in response to such mutations, despite the fact that these proteins are small and monomeric—precisely the category where AlphaFold is expected to perform best. Confidence metrics prove unreliable, as they select the most accurate structure at most 35% of the time and consistently correlate with the structural quality of the best available training-set template. ESMFold exhibits greater, though still imperfect, mutational sensitivity, suggesting a tighter coupling between sequence identity and predicted structure that may reflect differences in training objective rather than overall model quality. These findings indicate that AlphaFold may rely heavily on memorized templates rather than biophysical reasoning, with direct implications for mutation-effect interpretation, confidence-guided model selection, and sequence optimization workflows.

## Introduction

The release of AlphaFold 2 in 2021 fundamentally transformed structural biology [1–4]. By achieving near-experimental accuracy on a substantial fraction of protein targets, AlphaFold 2 demonstrated that deep learning could solve what had been considered one of biology’s grand challenges: predicting a 3-dimensional protein structure from its amino acid sequence alone [1,5,6]. This breakthrough catalyzed an explosion of research across computational biology, accelerating drug discovery, enabling structure-guided protein engineering, and providing structural insights into previously intractable systems [7–10].

AlphaFold 3, released in 2024, extended these capabilities further [11]. Beyond proteins, it can model nucleic acids, small molecules, and ligands, enabling prediction of complete biomolecular assemblies [11,12]. Its diffusion-based architecture and expanded training set yield improved accuracy, particularly for protein–protein interfaces and multimeric complexes [8,11,13,14]. As a result, AlphaFold 3 has rapidly become the de facto standard in computational structural biology, underpinning an expanding ecosystem of derivative tools and methods.

This ecosystem now extends far beyond structure prediction. Modern protein design frameworks such as RFdiffusion and ProteinMPNN rely on architectural principles and training strategies pioneered by AlphaFold [15–17]. Generative models for protein binder design—including BoltzGen, BindCraft, and variants of AlphaFold itself—use AlphaFold’s structure prediction modules as differentiable components through which gradients are backpropagated to optimize sequences [18–20]. Protein language models, which learn sequence representations without explicit structural supervision, are increasingly coupled with AlphaFold’s predictions to enable tasks ranging from fitness prediction to genomic design [21–23]. Tools for predicting binding affinity, protein–protein interactions, stability changes upon mutation, and functional annotations increasingly incorporate AlphaFold-derived features or use AlphaFold as a preprocessing step [23–26]. In experimental laboratories, AlphaFold predictions now routinely guide construct design, inform crystallization strategies, and aid in molecular replacement for structure determination [27–29].

The pervasive integration of AlphaFold into research workflows makes understanding its underlying capabilities—and limitations—a matter of broad scientific importance. If AlphaFold has learned the true biophysical principles that relate sequence to structure, then the tools built upon it inherit a robust foundation. However, if AlphaFold’s predictions are primarily driven by template matching to its library of training proteins—similar to a threading algorithm [30]—then derivative methods may be inheriting the same constraints and are effectively glued to the training structures regardless of what conformation the sequence of interest will actually adopt. The distinction is not trivial: Models that lack biophysical grounding are unlikely to generalize to novel protein families, engineered sequences far from natural distributions, or sequences with substantial mutations relative to characterized homologs [8,31–34].

There is mounting evidence that current structure prediction models struggle with such out-of-distribution inputs. AlphaFold’s performance degrades substantially on proteins with low sequence similarity to its training set [35,36]. Its predictions for alternative conformations and fold-switching proteins often fail to reflect known structural diversity or allostery, instead converging to single dominant folds [37–39]. When used for protein design, AlphaFold-based pipelines can generate sequences that appear well-folded in silico but fail to express, fold correctly, or exhibit intended functions in vitro [18,40,41]. These observations raise critical questions: To what extent has AlphaFold learned the biophysical rules governing protein folding? Does it reason about how specific residues contribute to stability, interface formation, and structural integrity? Or does it somehow recognize patterns from its training data and interpolate among known structures to essentially recapitulate the training structure?

Addressing these questions requires evaluating not just whether AlphaFold 3 is accurate, but whether its predictions respond to sequence perturbation in proportion to the expected structural impact of each mutation—a question of invariance rather than accuracy, and one that standard held-out benchmarks are not designed to answer [8,42–44]. Here, we perform a systematic adversarial evaluation of AlphaFold 3 using complementary point-mutation and deletion-mutation regimes, measuring deviation from the model’s own original prediction rather than from any experimental reference. We analyzed over 200 proteins spanning multiple categories, including proteins with and without training-set homologs and experimentally validated fold-switching proteins. We then compare AlphaFold 3 with ESMFold, a protein language model-based method that does not rely on multiple sequence alignments [45], and evaluate AlphaFold 3 confidence metrics in relation to those of AlphaFold 2.

To our knowledge, this study represents the first large-scale characterization of mutational invariance across both broad perturbation magnitudes and distinct protein/model classes. Specifically, no prior work has systematically evaluated structural invariance across mutation burdens ranging from 5% to 70% of the full sequence while simultaneously comparing these behaviors across fundamentally different folding architectures. Previous studies have reported limited sensitivity to small numbers of point mutations, but those observations were restricted in perturbation scope, with only a few point mutations introduced, often to measure ΔΔG rather than macroscopic structural changes [46–50]. By contrast, our framework systematically interrogates how structural predictions change under progressively severe adversarial perturbations across multiple biological and architectural contexts. Together, these analyses provide a direct test of whether AlphaFold 3 exhibits robust biophysical generalization or excessive structural invariance consistent with memorization-like behavior, with important implications for the reliability of AlphaFold-based protein design, drug discovery, and biological modeling workflows.

## Results

### Adversarial point-mutation analysis

To probe how AlphaFold 3 incorporates biophysical constraints and reality into its predictions, we conducted an adversarial point-mutational analysis. A total of 200 protein sequences spanning 4 categories—monomer-novel, monomer-similar, multimer-novel, and multimer-similar—were provided to AlphaFold 3 for structure prediction. Here, monomeric and multimeric denote the number of chains, while “similar” indicates greater than 30% sequence homology to at least one protein in the AlphaFold 3 training set, and “novel” indicates the absence of such homology. Each sequence was then mutated at 5%, 10%, 20%, 40%, and 70% point-mutation thresholds, with structure predictions generated for every mutated variant. Mutations were intentionally selected to be maximally disruptive—for example, substituting small hydrophobic residues with large polar residues—and were biased toward positions near the center of the sequence, a region more likely to map to the protein core [50,51]. A solvent-accessible surface area (SASA)-based weighting scheme targeting structurally buried residues directly was also tested and produced near indistinguishable results across all mutation levels and protein categories, as reported in the Supplementary Materials, so the simpler center-based heuristic was retained for computational efficiency. See Methods for more details.

Comparisons used both a global structural similarity metric, TM-score [52,53], and an interfacial accuracy metric, DockQ [53,54], as well as a superposition-based deviation metric, RMSD [55], and a local distance-based accuracy metric, lDDT [56], which captures residue-level structural agreement independent of global superposition.

**Fig. 1.**
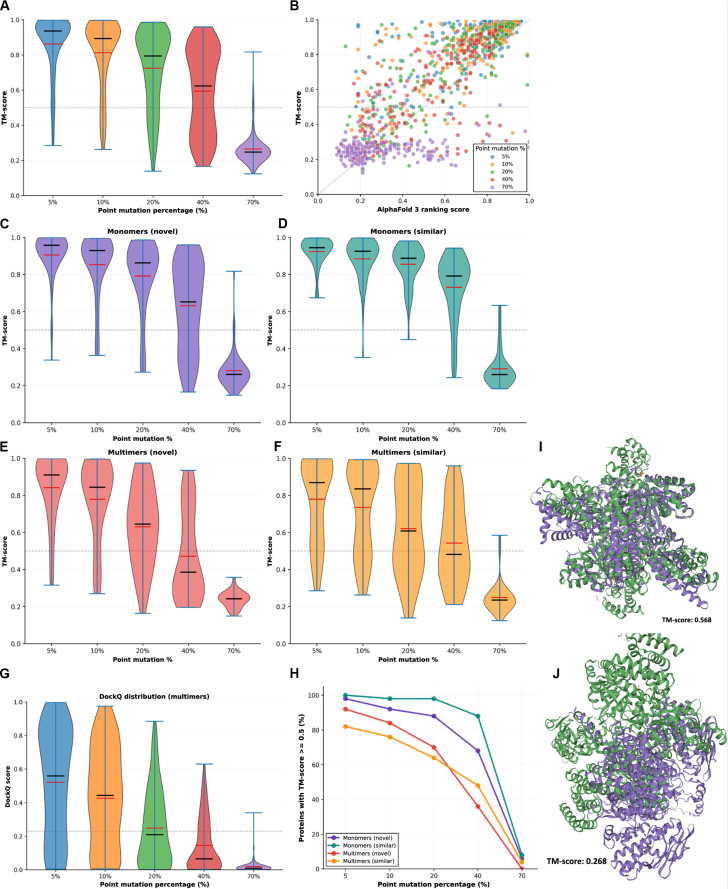
AlphaFold 3 exhibits structural invariance under adversarial point mutations. (A) Violin plots showing TM-score distributions for the full 200-protein dataset at each mutation threshold (5%, 10%, 20%, 40%, 70%), comparing mutated predictions to unmutated AlphaFold 3 predictions. (B) Correlation between AlphaFold 3 ranking confidence scores and structural accuracy (TM-score). Each point represents a single protein, colored by mutation percentage. (C to F) TM-score distributions stratified by protein category: (C) monomer-novel, (D) monomer-similar, (E) multimer-novel, and (F) multimer-similar. (G) DockQ score distributions for multimeric proteins (novel and similar bins combined), showing interfacial accuracy degradation across mutation thresholds. (H) Survival curve indicating the fraction of proteins maintaining accurate global fold (TM-score ≥ 0.5) at each mutation level. (I) Structural superposition of 7OCN with 40% mutation (purple) onto the unmutated prediction (green), showing preserved fold architecture (TM-score = 0.568). (J) Structural superposition of 7OCN with 70% mutation (purple) onto the unmutated prediction (green), showing fold destruction (TM-score = 0.268). All TM-scores and DockQ values reflect comparison to the unmutated AlphaFold 3 prediction rather than experimental structures.

As shown in Fig. 1, AlphaFold 3 maintains global fold similarity up to remarkably high levels of perturbation. Figure 1H presents a survival curve representing the fraction of structures that retain the same global fold—defined as TM-score ≥ 0.5—across mutation thresholds. At 40% mutation, monomeric proteins still maintain the same global fold on average, while multimeric proteins, though they degrade in quality more rapidly, still preserve global fold correctness in over 40% of cases. This pattern holds consistently for both novel and previously seen sequences, with no discernible difference observed between proteins novel to the AlphaFold 3 training set and those with homologs in it.

lDDT and RMSD analyses are reported in Table S6A. Although lDDT declines more rapidly than TM-score across mutation thresholds while still showing invariance, this primarily reflects degradation in local atomic packing rather than collapse of the overall fold, which remains comparatively stable by TM-score. RMSD also increases monotonically with mutation load, reaching 28.8 Å at 70% mutation, but RMSD is highly sensitive to local outliers, chain length, and cumulative deviations across multimeric complexes [52,53,57]. Accordingly, TM-score and DockQ remain the primary metrics for assessing structural conservation, as they more directly capture preservation of global fold and complex architecture.

The violin plots in Fig. 1A and G reveal how TM-score and DockQ distributions shift across mutation thresholds. TM-scores remain remarkably high through 70% mutation, declining only gradually as sequence identity erodes. In stark contrast, interfacial geometry degrades rapidly. DockQ scores show that by 20% mutation, approximately half of the interfacial structures are incorrect with respect to the structure predicted from the original unmutated sequence, highlighting that interfacial contacts are far more vulnerable to mutational perturbation than overall fold architecture.

AlphaFold 3’s internal confidence metrics show only moderate sensitivity to these perturbations. The relationship between mutation percentage and ranking score is modest, with a Pearson correlation coefficient of −0.558 and a Spearman rank correlation of −0.544. Consistent with this trend, the mean AlphaFold 3 ranking score decreases gradually from 0.73 at 5% mutation to 0.62 at 20%, and falls below the low-confidence regime only at the 40% mutation level. Notably, the commonly used threshold for high-confidence predictions is a ranking score of 0.6 [4,26]; thus, even at 20% mutation—where a substantial portion of the protein has been deliberately altered in a highly deleterious manner—the model remains confident in its predictions on average.

We extended the point-mutation analysis to 15 experimentally validated, monomeric fold-switching proteins compiled by a previous study [58]. For each protein, mutations were directed at residues or regions that prior work has empirically shown to induce fold-switching (details on the mutation regime for each protein are provided in Methods and in the Supplementary Materials). Table S1 summarizes the results. Consistent with the larger dataset, predicted structures remained highly invariant prior to the 40% mutation threshold, maintaining an average TM-score of 0.63 relative to the original predicted structure. Even for proteins where the empirically validated fold-switching residues or regions were purposefully mutated, AlphaFold 3 exhibited little detectable structural divergence, mirroring the behavior observed in non-fold-switching proteins. This result reinforces the conclusion that AlphaFold 3 frequently fails to capture biologically plausible mutational responses, even when multiple conformations are known to exist. The AlphaFold 3 ranking score similarly remains high up to the 40% threshold, further underscoring confidence metric invariance. Hence, even for the small proteins where fold-switching occurs, AlphaFold 3 does not reliably respond to known fold-switching mutations, suggesting that it should be used with caution in protein sequence optimization.

### Adversarial deletion mutation analysis

To further assess AlphaFold 3’s susceptibility to memorization or structural invariance, we conducted an analogous adversarial experiment using residue deletions, which are typically far more disruptive than point mutations [59]. Even a small number of deletions can cause severe destabilization or collapse of tertiary or quaternary structure [59,60]. Accordingly, lower deletion thresholds of 1%, 3%, 5%, and 10% were employed. As in the point-mutation experiment, deletions were biased toward centrally located residues to maximize structural disruption.

**Fig. 2.**
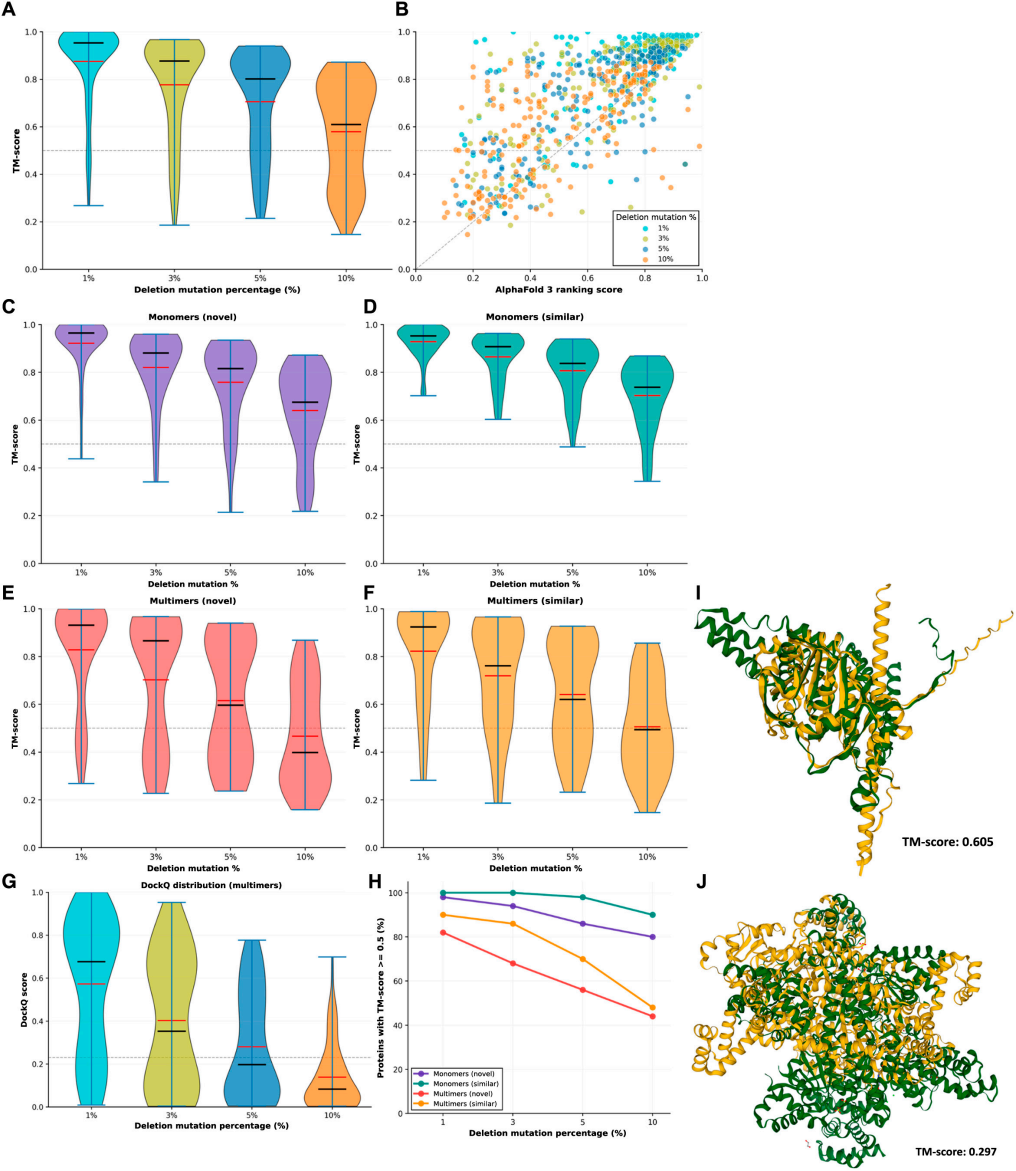
AlphaFold 3 exhibits structural invariance under adversarial deletion mutations. (A) Violin plots showing TM-score distributions for the full 200-protein dataset at each mutation threshold (1%, 3%, 5%, 10%), comparing mutated predictions to unmutated AlphaFold 3 predictions. (B) Correlation between AlphaFold 3 ranking confidence scores and structural accuracy (TM-score). Each point represents a single protein, colored by mutation percentage. (C to F) TM-score distributions stratified by protein category: (C) monomer-novel, (D) monomer-similar, (E) multimer-novel, and (F) multimer-similar. (G) DockQ score distributions for multimeric proteins (novel and similar bins combined), showing interfacial accuracy degradation across mutation thresholds. (H) Survival curve indicating the fraction of proteins maintaining accurate global fold (TM-score ≥ 0.5) at each mutation level. (I) Structural superposition of 8CQZ at 10% mutation (yellow) onto the unmutated prediction (green), showing preserved fold architecture (TM-score = 0.605). (J) Structural superposition of 7OCN at 10% deletion (yellow) onto the unmutated prediction (green), showing original fold destruction (TM-score = 0.297). All TM-scores and DockQ values reflect comparison to the unmutated AlphaFold 3 prediction rather than experimental structures.

Despite the severity of this perturbation type, AlphaFold 3 again maintains global fold integrity, as shown in Fig. 2. The survival plot in Fig. 2H captures this clearly: Even at 10% deletions, an overwhelming majority of monomeric proteins retain accurate predicted structures. Multimeric proteins decrease in accuracy more rapidly than monomers, consistent with the point-mutation results, but no substantial difference was observed between novel and similar proteins within either category.

The violin plots in Fig. 2A and G illustrate TM-score and DockQ distributions across deletion thresholds. The relatively modest decrease in structural accuracy under deletion mutations—compared to point mutations—reflects the lower maximum mutation level tested. Nevertheless, the pattern of fold preservation is striking given that deletions near the protein core would be expected to cause substantial structural rearrangement or complete loss of fold in real proteins. The structural accuracy scores decline more steeply for multimeric proteins, crossing into low-confidence regimes by the 5% deletion threshold. Monomeric proteins, however, preserve high correspondence to the original protein structure across all tested deletion levels.

lDDT and RMSD analyses are reported in Table S6B, and confirm the same pattern observed under point mutations—lDDT degrades slightly more rapidly than TM-score across deletion thresholds, indicating that local atomic arrangement is more sensitive to residue removal than global fold topology.

Furthermore, AlphaFold 3’s internal ranking score exhibits increased sensitivity under deletion-based perturbations, yet still displays a notable degree of invariance relative to the severity of the structural disruption. The association between deletion fraction and ranking score is moderate, with a Pearson correlation coefficient of −0.412 and a Spearman rank correlation of −0.443. The mean ranking score declines steadily as the proportion of deleted residues increases, decreasing from 0.76 at 1% deletions to 0.68 at 3%, 0.60 at 5%, and 0.48 at 10%. While this downward trend is more pronounced than in the point-mutation experiment, the model’s confidence remains relatively high at low deletion levels even though sparse deletions—particularly within the protein core—would be expected to substantially impair structural integrity in real proteins [61,62]. This persistence of elevated confidence scores mirrors the observed invariance in predicted structural accuracy.

### Comparison of AlphaFold 3 and ESMFold under adversarial mutation

To determine whether the structural invariance observed under mutation is specific to AlphaFold 3 or reflects a broader property of deep learning-based structure prediction, we compared AlphaFold 3 with ESMFold across the same mutational regimes. Because ESMFold is not designed to process multimeric inputs, this comparison was restricted to monomeric proteins, and AlphaFold 3’s monomeric predictions were used accordingly.

**Fig. 3.**
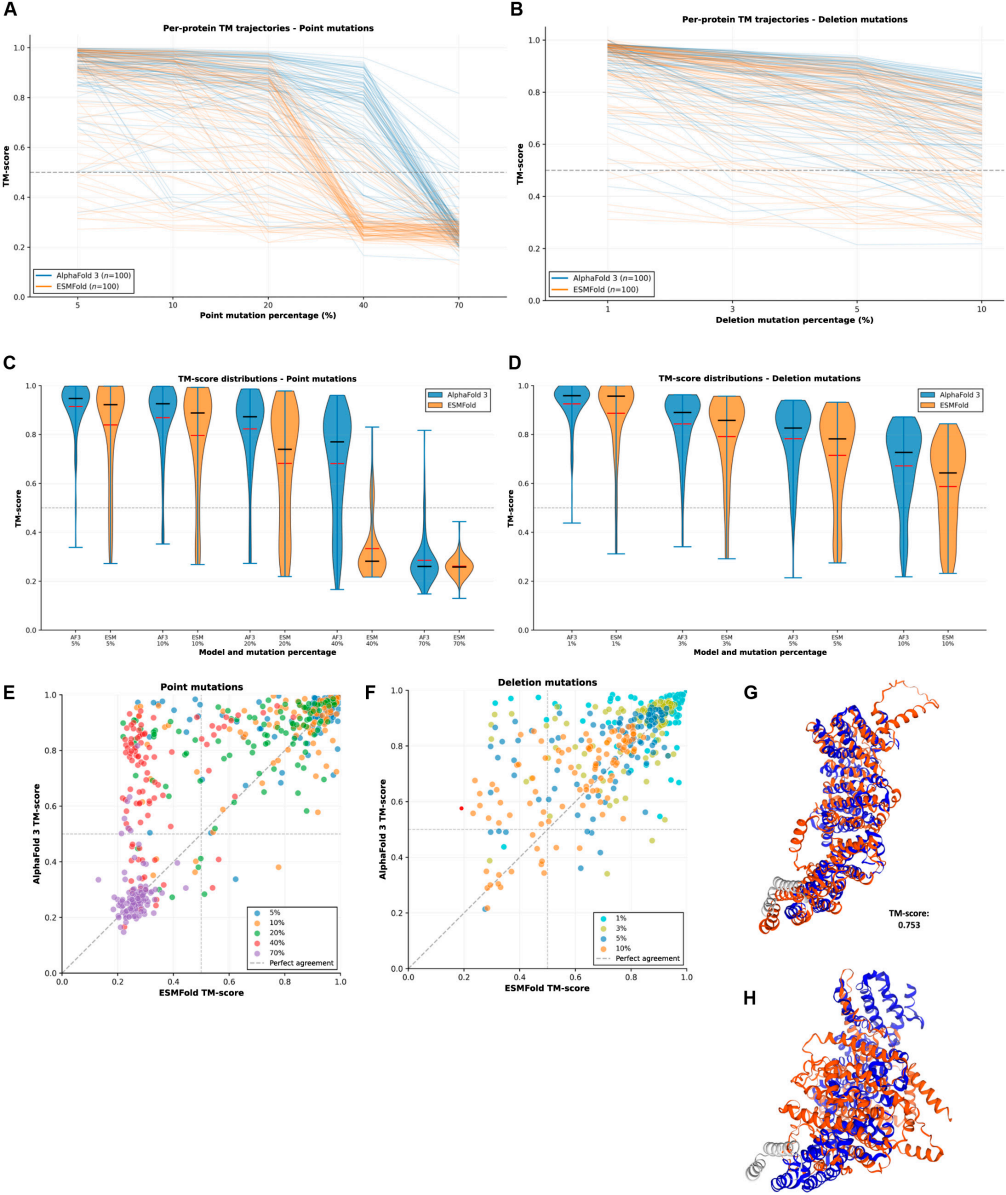
ESMFold exhibits greater sensitivity to point mutations than AlphaFold 3. (A) Individual protein accuracy trajectories under point mutations. Each line represents a single protein, with ESMFold (orange) and AlphaFold 3 (blue) predictions compared to their respective unmutated structures. (B) Individual protein accuracy trajectories under deletion mutations, colored as in (A). (C) TM-score distributions across point-mutation thresholds for ESMFold (orange) and AlphaFold 3 (blue). (D) TM-score distributions across deletion mutation thresholds, colored as in (C). (E) Direct comparison of model accuracy under point mutations. Each point represents a protein at a given mutation threshold, with color indicating mutation percentage. Points above the diagonal indicate greater accuracy for AlphaFold 3. (F) Direct comparison of model accuracy under deletion mutations, displayed as in (E). (G) Structural superposition of AlphaFold 3 predictions for protein 8RQT: 40% mutated structure (red) aligned to unmutated prediction (blue), demonstrating structural preservation (TM-score = 0.753). (H) Structural superposition of ESMFold predictions for protein 8RQT: 40% mutated structure (red) aligned to unmutated prediction (blue), demonstrating original fold destruction (TM-score = 0.293). ESMFold shows substantially greater structural divergence at intermediate mutation levels, suggesting higher sensitivity to sequence perturbations. All comparisons are between mutated and unmutated predictions from the same model.

Figure 3 presents the results of this analysis. Figure 3A and B shows the individual trajectories of each protein in terms of TM-score accuracy under point mutations and deletion mutations, respectively. Under deletion mutations, ESMFold and AlphaFold 3 perform comparably, with ESMFold exhibiting consistently greater flexibility—that is, lower structural similarity to its original prediction—across all deletion thresholds. This suggests that ESMFold’s predictions are slightly more sensitive to the disruptive effects of residue removal. Whether they yield experimentally correct structures after mutation is still an open question.

The contrast is far more pronounced for point mutations, however. Between 20% and 40% point mutation, ESMFold’s accuracy with respect to its original predicted structure declines quickly and starkly, while AlphaFold 3 maintains its trend of gradual decrease. It is not until 70% mutation—corresponding to only 30% sequence identity—that AlphaFold 3 exhibits a comparable collapse, catching up to ESMFold’s degraded performance. This pattern is also evident in Fig. 3E, a scatterplot demonstrating that ESMFold performance falls substantially below that of AlphaFold 3 at intermediate mutation levels. lDDT and RMSD analyses reported in Table S6C further support this conclusion, with both metrics degrading far more rapidly for ESMFold than for AlphaFold 3 between the 20% and 40% mutation thresholds.

We also applied ESMFold to the same 15 fold-switching proteins analyzed with AlphaFold 3 (Table S1). Consistent with the broader 200-protein dataset, ESMFold exhibited greater sensitivity to point mutations, with predicted structures diverging substantially from the original prediction beyond the 10% mutation threshold—though the effect was less pronounced than in the full dataset.

These results indicate that ESMFold is more sensitive to deleterious point mutations than AlphaFold 3 across the mutation regimes tested. While both models eventually converge at extreme mutation levels, ESMFold’s earlier response to sequence perturbation is consistent with its architecture: As a single-sequence model trained with a masked language modeling (MLM) objective, ESMFold learns sequence–structure relationships purely from residue covariation statistics without access to multiple sequence alignments or explicit structural templates [45]. This is not to suggest that ESMFold is a superior structure prediction tool—on the contrary, it achieves substantially lower absolute accuracy than AlphaFold 3 on standard benchmarks [29,42,43]. Rather, its training objective may instill a tighter coupling between sequence identity and predicted structure such that biophysically disruptive mutations more immediately produce structural responses.

### Evaluation of confidence metrics in AlphaFold 2 versus AlphaFold 3

To assess how reliably AlphaFold 2 and AlphaFold 3 estimate the accuracy of their own predictions, we evaluated both systems on 100 proteins drawn from the monomer-novel and multimer-novel bins [1,11]. This restriction avoids giving AlphaFold 3 an advantage from its broader training distribution and ensures a fair comparison [8]. Predicted structures were compared against experimentally determined structures from the Protein Data Bank (PDB), rather than against the original unmutated predictions used in the adversarial analyses above.

**Fig. 4.**
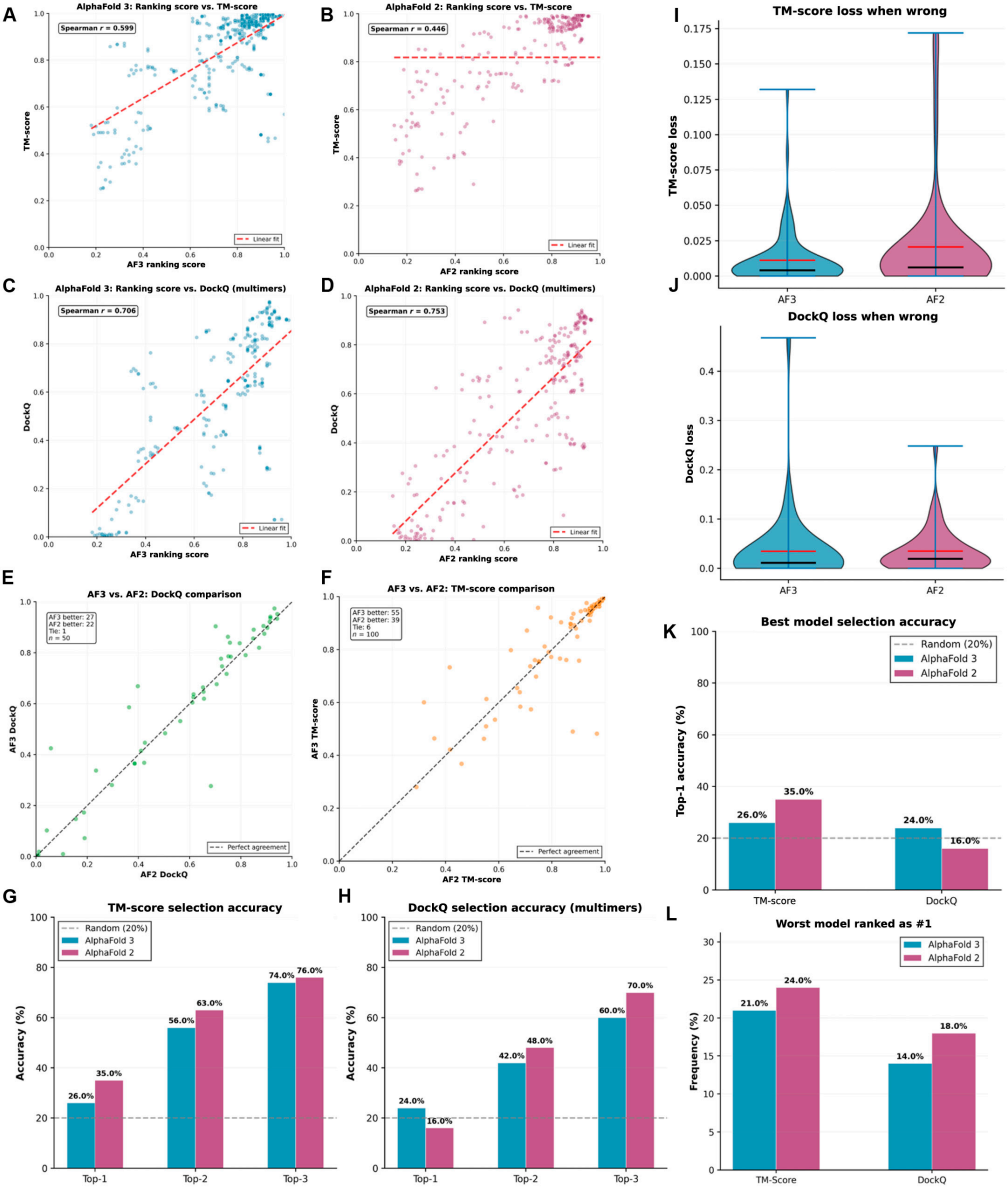
AlphaFold 2 and AlphaFold 3 confidence metrics fail to reliably identify the most accurate predictions. (A to D) Correlation between ranking confidence scores and structural accuracy for AlphaFold 3 (A and C) and AlphaFold 2 (B and D). TM-score is shown for monomers (A and B) and DockQ for multimers (C and D). Spearman correlation coefficients are indicated, with lines of best fit shown in red. (E and F) Direct comparison of structural accuracy between AlphaFold 2 and AlphaFold 3 on novel proteins, measured by (E) TM-score (monomers and multimers) and (F) DockQ (multimers only). Points above the diagonal indicate superior AlphaFold 3 performance. (G) Frequency with which each model selects the most accurate structure (rank 1), or a structure within the top 2 or top 3 most accurate, based on TM-score across 100 proteins. AlphaFold 3 (blue) and AlphaFold 2 (purple). (H) Model selection accuracy based on DockQ for 50 multimeric proteins, displayed as in (G). (I) Distribution of TM-score loss incurred when the model-selected structure is not the most accurate prediction. Larger values indicate more severe selection errors. (J) Distribution of DockQ loss incurred by incorrect model selection, displayed as in (I). (K) Combined view of model selection accuracy for both TM-score and DockQ metrics, showing the percentage of proteins for which each model correctly identifies the best prediction. (L) Frequency with which each model selects the least accurate (worst) prediction, highlighting severe ranking failures. Both models exhibit poor calibration, selecting the optimal structure in only 16% to 35% of cases.

As shown in Fig. 4, AlphaFold 3 achieves statistically significantly higher accuracy than AlphaFold 2, particularly for multimeric complexes. This improvement has been noted in prior work [8,11], but another critical question is whether either model can reliably identify which of its generated predictions is most accurate. Standard practice is to generate 5 candidate structures and select the top-ranked prediction based on internal confidence metrics.

Figure 4K reveals the limitations of both models’ confidence-based selection. AlphaFold 3 selects the most accurate structure—by TM-score or DockQ—only approximately 25% of the time, while AlphaFold 2 achieves at most 35%. Even when the definition of success is relaxed to include selecting among the top 2 or 3 most accurate structures, as shown in Fig. 4G and H, accuracy increases but never exceeds 80% for either model.

Incorrect model selection is not inconsequential. On average, selecting the wrong model costs approximately 0.01 TM-score points for AlphaFold 3 and 0.02 for AlphaFold 2. DockQ loss averages approximately 0.03 for both models. However, in some instances, the losses are far more severe: up to 0.17 for TM-score and over 0.40 for DockQ. Figure 4I and J illustrates that choosing the wrong model is not always a harmless error—substantial accuracy can be forfeited when confidence metrics fail to identify the best prediction.

We also evaluated the correlation between each model’s ranking score—a linear combination of ipTM if applicable, pTM, and clash penalties [11]—and structural accuracy against the ground truth. The Spearman correlation coefficient was higher for AlphaFold 2 on multimeric proteins but higher for AlphaFold 3 on monomeric proteins, as shown in Fig. 4A to D. The linear relationship between predicted score and accuracy, quantified by the Pearson correlation coefficient, was stronger for multimers than for monomers in both models. For multimers, the correlations were 0.72 for AlphaFold 3 and 0.83 for AlphaFold 2, indicating greater sensitivity of AlphaFold 2’s ranking score in this setting. In contrast, for monomers, the correlations were 0.59 for AlphaFold 3 and 0.44 for AlphaFold 2, suggesting superior performance of AlphaFold 3 on monomeric predictions. These results indicate that while confidence metrics provide useful signal, they remain imperfect guides for selecting the most accurate prediction—a limitation with practical implications for downstream applications that depend on model-selected structures.

### Structural template availability varies with AlphaFold 3 confidence

Having established that AlphaFold 3’s structural predictions are resistant to mutation, we asked a more pointed question: What do its confidence metrics measure beyond that? If the model’s uncertainty estimates reflect genuine biophysical reasoning, then they should be insensitive to factors external to the sequence itself. If instead they reflect template availability, confidence becomes a proxy for how well-covered the query is in the training set rather than a signal of prediction quality. In an effort to distinguish these possibilities, we performed an exhaustive structural search of the pre-cutoff PDB using FoldSeek [63], identifying for each of the 200 proteins in our dataset the most structurally similar chain deposited before the AlphaFold 3 training cutoff, and directly quantified its relationship to model confidence.

The result is notable. AlphaFold 3’s ranking score correlates significantly with the query TM-score of the best pre-cutoff structural template (Pearson r=0.39, $P=7.8\times 10^{-5}$; Spearman ρ=0.39, $P=8.2\times 10^{-5}$), suggesting that the model is more confident when a structurally similar training exemplar exists. By contrast, sequence identity to that same template is a weak and nonsignificant predictor (r=0.033, P=0.744), indicating that confidence tracks structural rather than sequential similarity to training-set exemplars. A comparable, though weaker, effect holds for multimers (r=0.27, $P=7.7\times 10^{-3}$; ρ=0.30, $P=3.1\times 10^{-3}$).

Stratifying monomers by novelty reveals that the correlation is robust regardless of whether the query sequence appears in the training distribution—novel proteins (r=0.51, $P=1.9\times 10^{-4}$) and similar proteins (r=0.49, $P=3.1\times 10^{-4}$) show comparable effect sizes despite differing substantially in their template landscapes (Table S9). For multimers, the correlation is significant among novel complexes (r=0.43, $P=2.2\times 10^{-3}$) but absent for similar complexes (r=0.14, P=0.34), which may suggest that sequence memorization plays a larger role when the query falls within the training distribution, though the precise mechanism warrants further investigation.

For monomers, the single best structural template is the strongest predictor of confidence (Spearman ρ=0.35, $P=4.4\times 10^{-4}$), with correlations declining for deeper neighborhoods, and neighborhood density offering no additional predictive value. Multimers exhibit the opposite trend: Correlations increase monotonically from ρ=0.30 at the top hit to ρ=0.46 at the top 100 hits ($P=2.3\times 10^{-6}$), consistent with the possibility that for complex prediction, breadth of structural coverage in the training set contributes more to model confidence than any single exemplar. Taken together, these findings are consistent with the interpretation that AlphaFold 3’s confidence estimates partly reflect training-set familiarity, a pattern with potential consequences for workflows that rely on ranking scores to guide downstream decisions. See Table S9 for additional details.

## Discussion

The point and deletion mutation analyses presented here reveal a striking invariance in both the structures predicted by AlphaFold 3 and in its confidence metrics under substantial sequence perturbations. Using the commonly accepted TM-score threshold of 0.5 to indicate similar folds [53], we observed that up to 40% of residues in a protein can be mutated—and up to 10% deleted—before the predicted structure meaningfully diverges from the original AlphaFold 3 prediction on average. This trend persists even for fold-switching proteins, which are experimentally known to adopt multiple conformations, highlighting a verifiable disconnect between model predictions and expected biological behavior [58].

These observations have important implications for how the field interprets and applies structure prediction models. While previous work has noted that AlphaFold exhibits some insensitivity to point mutations, the extent of this phenomenon warranted closer examination [32]. Insensitivity to 1 or 2 substitutions may be expected, as minor perturbations often leave the global fold intact. However, changing up to 70% of the amino acids in an intentionally deleterious manner while still observing the same predicted global fold raises fundamental questions about the learned sequence-structure mapping.

It is critical to note that our mutation strategy was not designed to mimic natural evolutionary divergence, in which substitutions are biophysically filtered by selection, but to test adversarial and unphysical mutations deliberately incompatible with the native fold. These mutations are not analogous to structurally similar yet sequentially distant homologs; such homologs represent constrained evolutionary trajectories rather than the random, maximally disruptive perturbations utilized here. We acknowledge that the structural consequences of our mutations were not experimentally verified, and it is conceivable that a small number of low-burden mutations are genuinely tolerated by certain proteins. However, this is highly unlikely to account for the invariance observed across 200 proteins at mutation loads of 20% to 70% under purposefully deleterious mutations. Given the fold invariance demonstrated by AlphaFold 3 under such extreme conditions, which should yield nonphysical structures, AlphaFold 3 cannot be used to measure the structural consequences of more modest mutational changes as would occur under natural selection.

One interpretation of these results is that AlphaFold 3 relies heavily on identifying similarities to known templates rather than reasoning from biophysical principles, consistent with studies showing that AlphaFold over-relies on its training set when predicting alternative conformations [37]. This is also consistent with the broader literature on learned representations in protein engineering, which has highlighted the difficulty of disentangling genuine biophysical reasoning from pattern matching in deep learning models [37,50,64–66]. Thus, caution should be exercised when AlphaFold 3 is a major component in protein design algorithms.

A related limitation concerns conformational dynamics and allostery. AlphaFold 3 produces a single static structure and does not model conformational ensembles or allosteric effects by which conformational changes at one site modulate function at a distal site [67–73]. Because AlphaFold 3 fails to register even gross structural consequences of severe sequence perturbations, it is unlikely that it captures the subtler conformational shifts that underlie allosteric communication—a limitation particularly consequential for drug discovery and protein engineering applications where allosteric modulation is a central design objective [74,75].

The comparison with ESMFold provides additional context on the model specificity of these concerns. ESMFold exhibits greater sensitivity to point mutations than AlphaFold 3, consistent with research demonstrating that ESM models learn coevolutionary statistics through MLM [76]. While ESMFold achieves lower absolute accuracy for structure prediction, its greater responsiveness to sequence perturbation is also evident for fold-switching proteins. This is not to suggest that ESMFold’s sensitivity necessarily reflects correct structural responses to mutation. Rather, the contrast suggests that input modality and learning objective shape how invariant model prediction is to sequence mutations.

These findings also underscore concerns about confidence metric reliability. Neither AlphaFold 2 nor AlphaFold 3 reliably identifies the most accurate structure among 5 generated predictions, with both selecting the best structure only 16% to 35% of the time. Previous studies have documented issues with AlphaFold confidence metrics [41,46,48,77], and modifications to confidence calculations do not fully address the underlying bias [8]. Despite a more sophisticated atomic-level confidence module, AlphaFold 3’s model selection accuracy differs only modestly from AlphaFold 2 [1,11], suggesting that the limitation is fundamental rather than implementation-specific. The structural template analysis supports this: AlphaFold 3’s confidence correlates significantly with the structural quality of the best pre-cutoff template, indicating that uncertainty estimates reflect template availability more than genuine prediction quality.

The practical consequences merit consideration. When an incorrect model is selected, losses in accuracy can be substantial—reaching up to 0.17 in TM-score and over 0.4 in DockQ—and for applications in which predictions inform downstream decisions, these errors could lead to the exclusion of promising candidates or the retention of problematic ones [15,18]. AlphaFold 3’s structural invariance carries additional implications for gradient-based design pipelines, such as BoltzGen and BindCraft [18,20]: When predicted structures do not respond to sequence perturbation, gradients backpropagated through the model carry no meaningful sequence–structure signal. Recent work demonstrates that AlphaFold-based pipelines produce high-confidence candidates that fail experimentally at nontrivial rates [41], and our findings suggest a plausible contribution to this failure mode.

Nevertheless, these observations do not diminish the transformative impact of AlphaFold on structural biology. For the majority of proteins and applications, and particularly the structural modeling of stable wild-type proteins, AlphaFold 3 achieves remarkable accuracy and has enabled research that would otherwise be impractical. The limitations we document are most relevant for out-of-distribution cases and applications requiring sensitivity to sequence variation such as mutation-effect interpretation, confidence-guided model selection, and sequence optimization.

The source of these limitations remains an open question. One possibility is architectural: transformer and diffusion-based frameworks may inherently favor interpolation within the training distribution. A second lies in the training data, which consists almost exclusively of stable wild-type proteins, meaning that, without exposure to destabilizing mutants, models may never learn the relationships necessary to predict mutation-induced disruption. ESMFold’s greater sensitivity is consistent with this view, suggesting that a pretraining objective similar to the MLM objective of ESMFold may help AlphaFold models improve their input sequence and predicted structure coupling. A third possibility is that models operate within an inherent radius of convergence, responding only when mutation load escapes the local structural basin. A fourth is that the MSA partially buffers mutational sensitivity by providing evolutionary context that compensates for changes in the query sequence—a dynamic supported by recent work demonstrating that removing MSA input entirely causes structural accuracy to collapse even for unmutated sequences [78,79], suggesting that the relationship between single-sequence and alignment-derived information is more complex than it may appear. The precise mechanistic attribution among these possibilities remains an important open question for future work.

Several directions may help address these limitations. Training on datasets that include destabilized mutants and misfolded proteins could expose models to a broader range of sequence-structure relationships, and expanding conformational diversity in training data—incorporating multiple experimentally determined conformers per protein rather than a single representative structure—could enable models to learn embeddings that simultaneously capture the full conformational landscape a sequence can adopt [37,80]. Incorporating explicit biophysical priors into model architectures or loss functions may enforce more realistic mutational responses [50,81]. Developing better-calibrated confidence estimation methods would enhance the reliability of model-guided decisions, particularly for out-of-distribution sequences where current ranking scores appear to reflect template availability more than genuine prediction quality [8,82]. At the practical level, cross-validation between AlphaFold 3 and ESMFold predictions provides an immediately actionable consistency check for sequence optimization workflows. For lower-order mutations, these predictions can be further supplemented with structure-based stability predictors trained on experimental stability measurements, such as ThermoMPNN and SPURS [83,84]. Finally, continued benchmarking against adversarial and edge cases will be essential for characterizing the boundaries of model applicability.

The remarkable successes of AlphaFold should not obscure the work that remains. Structure prediction has advanced enormously, but the ability to reliably predict how arbitrary proteins—including those with extensive mutations or novel sequences—will fold remains incomplete. Recognizing the current limitations of these tools is a necessary step toward developing the next generation of methods that more fully capture the relationship between protein sequence and structure.

## Methods

### AlphaFold testing dataset collection

To rigorously evaluate AlphaFold’s performance, we assembled a dataset of 200 proteins, divided evenly into 4 categories: multimer-novel, multimer-similar, monomer-novel, and monomer-similar, with 50 proteins per category. “Multimeric” and “monomeric” indicate whether a protein contains multiple chains or a single chain, respectively, while “novel” and “similar” denote sequence similarity to proteins in the PDB released on or before 2021 September 30 [11,85]. Proteins labeled as similar share 30% or more sequence homology with pre-September 2021 PDB entries, meaning that their homologs could plausibly have been in AlphaFold 3’s training set [8]. In contrast, novel proteins have no such homology. Importantly, all proteins in the dataset were released after 2021 September 30, and no 2 proteins share 30% or more sequence similarity, ensuring minimal redundancy and that none were present in AlphaFold 3’s training set. This categorization enables a detailed assessment of AlphaFold’s performance across different protein types and levels of novelty.

### Fold-switching protein dataset

To extend the mutational analysis to proteins with experimentally validated alternative conformations, we selected 15 fold-switching proteins from the curated dataset compiled by Porter and Looger [58]. All proteins in this set are monomeric and have been experimentally confirmed to adopt multiple distinct folds under different conditions. The selected proteins correspond to the following PDB entries: 2KXO, 2LSH, 4OV8, 2MZ7, 4PMK, 2N4O, 2KTM, 2LE3, 2X9C, 3J9E, 5SUZ, 4HLS, 1S5P, 3TKA, and 3GAX. For these proteins, only chain A from their corresponding PDB files was used, as described in [58]. Point mutations were introduced to these proteins using procedures similar to those applied to the 200-protein dataset described above, with the exception of a modified position-weighting scheme that prioritized residues or regions known to induce fold-switching. Details of this scheme are provided in Table S7. MSA processing and AlphaFold 3 inference followed the same protocols, with templates excluded. This analysis allowed us to assess whether proteins known to exhibit conformational plasticity show greater structural responsiveness to mutations in AlphaFold 3’s predictions compared to proteins without documented fold-switching behavior.

### Mutation strategy

The mutation percentages applied differed between point mutations and deletion mutations to reflect their relative biophysical severity. Point mutations were tested at 5%, 10%, 20%, 40%, and 70% thresholds, while deletion mutations were tested at 1%, 3%, 5%, and 10% thresholds. This asymmetry reflects the fact that deletion mutations are substantially more deleterious than point mutations [59]. Even a single residue deletion in a critical region can cause severe structural destabilization or complete loss of fold, whereas point mutations—particularly conservative substitutions—are often tolerated without catastrophic consequences [60]. By using lower deletion percentages, we ensured that the perturbations remained within a regime where structural prediction could be meaningfully evaluated while still probing the model’s sensitivity to this severe class of mutation.

Mutations were applied cumulatively across all thresholds. Each higher mutation percentage retained all mutations from the previous level and added new mutations to reach the target threshold. For example, the 20% point-mutation configuration contained all mutations present in the 10% configuration, plus additional mutations to reach 20% of the sequence length. Similarly, the 5% deletion configuration included all deletions from the 3% level, supplemented with further deletions. This cumulative strategy ensures that differences in structural predictions across mutation levels reflect the incremental addition of perturbations rather than independent sampling at each threshold, enabling a more controlled assessment of mutational sensitivity.

For homomeric complexes, mutations were applied synchronously: The same residue positions were mutated to the same amino acids across all identical chains. For example, in a homodimeric protein, if position 42 was mutated from serine to tryptophan in chain A, position 42 was also mutated from serine to tryptophan in chain B. This approach reflects the biological reality that homomeric complexes consist of identical gene products and thus share identical sequences. In contrast, heteromeric complexes have distinct chains with independent sequences, and mutations were applied independently to each chain such that different positions and amino acid substitutions were selected for each unique chain type.

### AlphaFold model configurations and inputs

For all mutational analyses, AlphaFold 3 was run using its default configuration, with 10 recycling steps and 5 diffusion samples [11]. The primary modifications were applied to the input features. Template models—which AlphaFold 3 uses to infer general structural information and which could artificially inflate mutational invariance—were excluded [11]. For both deletion and point mutations, the same random seed was used across all mutation thresholds to ensure comparability of model accuracy. All default settings were used for MSA generation in AlphaFold 3.

In the deletion mutation regime, multiple sequence alignments (MSAs) were modified by removing columns corresponding to deleted residues (see Algorithm 2 for pseudocode). This ensured compatibility with AlphaFold 3, which cannot process MSAs containing sequences whose lengths differ from that of the input sequence. In contrast, for the point-mutation regime, MSAs were left unchanged and only the input sequences were mutated, leveraging the fact that MSAs naturally encode organismal sequence variation (see Algorithm 1 for pseudocode).

For deletion mutations, the paired MSA was excluded from the input data. Previous studies have shown that paired MSAs do not improve structural prediction accuracy; thus, they were omitted to simplify the required modifications.


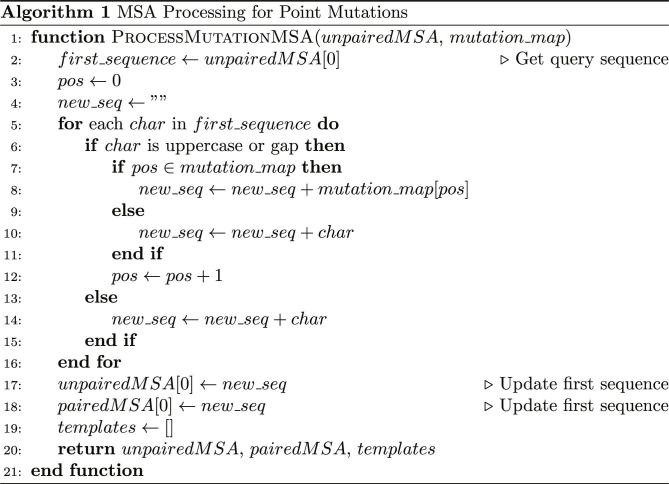


To verify that modifying MSAs—rather than regenerating them after mutation—did not introduce artificial mutational invariance, MSAs were fully regenerated for the same 200 proteins in the dataset using AlphaFold 3’s standard MSA generation pipeline. These proteins were first mutated at all tested thresholds (5%, 10%, 20%, 40%, and 70% for point mutations; 1%, 3%, 5%, and 10% for deletions), and then new MSAs were generated de novo for each mutated sequence using the default AlphaFold 3 MSA generation pipeline. The paired MSA was retained in this regeneration experiment, as it was accurately generated for the mutated sequences. No structural templates were used. No major differences in TM-score or AlphaFold 3 ranking score between models using modified versus regenerated MSAs were noted. Interestingly, models with regenerated MSAs exhibited slightly greater mutational invariance than their directly modified counterparts, suggesting that MSA regeneration only exacerbates the model’s structural invariance.

For the comparative analysis between AlphaFold 3 and AlphaFold 2, both models were configured with 10 recycles and 5 samples [1,11]. AlphaFold 3 was run without templates to ensure that its template-based features, which are absent in AlphaFold 2, did not confer an unfair performance advantage. As noted in the Results section, this analysis was conducted on 100 proteins drawn from novel bins—50 monomers and 50 multimers—which had no homologs in the AlphaFold 3 training set.


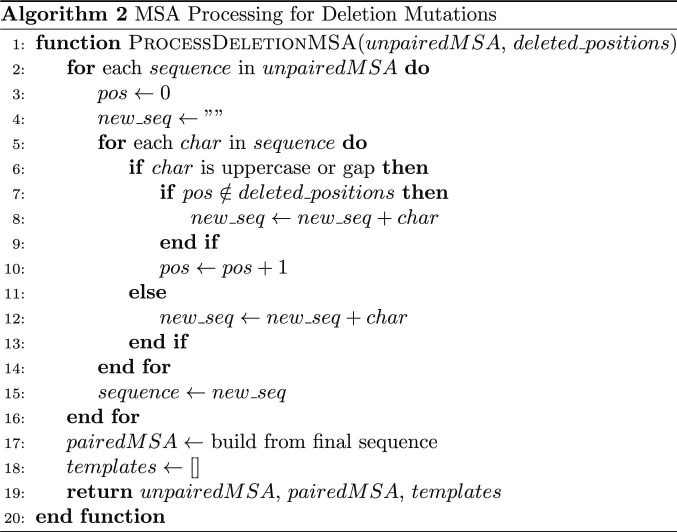


### Protein mutation details

The point mutations applied to proteins in the 200-protein dataset were designed to be as deleterious as possible, ensuring that the mutations were unlikely to incidentally enhance structural stability. The mapping of these mutations is summarized in Table S8. To further increase the likelihood of major structural disruption, the residue mutation algorithms for both point and deletion mutations incorporated a positional bias that preferentially selected residues near the center of the protein [51]. This bias was implemented mathematically by assigning each residue i a weight

$$w_{i}=\epsilon +\left(1-\epsilon \right)\left[\;1-\frac{|i-\left(L-1\right)/2|}{\left(L-1\right)/2}\;\right],\;i=0,1,\ldots ,L-1$$ (1)

where L is the sequence length and ϵ defines the weight for residues at the sequence termini, which, for the purposes of the study, was set to 0.1. This scheme ensures that central residues, which are more likely to contribute to the protein’s core stability, have a higher probability of mutation.

### ESMFold configuration and analysis

To compare AlphaFold 3’s mutational sensitivity with that of an alternative deep learning-based structure prediction model, we performed parallel analyses using ESMFold [45,76]. The ESMFold implementation was obtained from the HuggingFace [86,87] repository and run with all default configuration settings. Because ESMFold was not trained to predict multimeric protein structures, this comparison was restricted to monomeric proteins only [45]. Specifically, we analyzed the 100 monomeric proteins from the 200-protein dataset, comprising 50 from the monomer-novel bin and 50 from the monomer-similar bin. Point mutations and deletion mutations were applied to these proteins using the identical mutation strategy employed for AlphaFold 3. For each mutated protein, ESMFold predictions were compared to the original unmutated ESMFold prediction.

### FoldSeek exhaustive structural search

Each of the 200 experimentally determined structures was queried against FoldSeek’s PDB100 database [63], which comprises approximately 340,000 representative chains clustered at 100% sequence identity from the full PDB. Searches were conducted in exhaustive mode (–exhaustive-search 1) with TM-align-based scoring (–alignment-type 1), bypassing FoldSeek’s heuristic prefilter to guarantee globally optimal structural matches, yielding approximately 9,800 hits per query. Results were post-filtered to retain only hits deposited on or before 2021 September 30, using a list of approximately 180,000 pre-cutoff PDB identifiers obtained from RCSB PDB [85]. The query-normalized TM-score (qtmscore) was used as the primary structural similarity metric, as normalizing by query length prevents partial domain matches from inflating similarity estimates.

For monomeric proteins, AlphaFold 3’s monomeric ranking score, pTM, was used as the confidence metric; for multimeric complexes, the ranking score (0.8×ipTM+0.2×pTM) was used [11], jointly capturing fold accuracy and interface quality. For monomers, the top 100 pre-cutoff hits were extracted per query and ranked by qtmscore. One monomer had no pre-cutoff hits, yielding n=99 for the primary correlation analyses; 3 further monomers had fewer than 100 pre-cutoff hits, yielding n=96 for the neighborhood density analysis. For multimeric complexes, FoldSeek’s monomer search mode decomposed each complex into constituent chains, which were searched independently. Per-chain neighborhood statistics were computed from the top 100 pre-cutoff hits and averaged across chains to obtain complex-level estimates. Two multimers were excluded because at least one chain had fewer than 100 pre-cutoff hits, yielding n=98 used throughout. Minimum-across-chains and chain-length-weighted aggregation schemes were also tested and produced comparable results. Only mean aggregation is reported for simplicity.

### Structure comparison metrics

TM-score, lDDT, RMSD, and DockQ were calculated using the OpenStructure package [88]. TM-score quantifies global structural similarity between 2 protein structures and is normalized to a range of 0 to 1, where values above 0.5 typically indicate the same fold [52]. DockQ assesses the quality of predicted protein–protein interfaces by combining information about interface RMSD, ligand RMSD, and native contact fraction [54]. For multimeric proteins with more than 2 chains, the weighted average DockQ score across all pairwise chain interfaces was computed and used as the reported metric, with a standard threshold of 0.23 used for a valid interfacial conformation [54,89]. Both metrics were calculated by comparing mutated predictions to the original unmutated AlphaFold 3 prediction for the mutational invariance analyses, or to experimental structures from the PDB for the model comparison analyses.

Critically, the mutational invariance analyses compare mutated predictions against the model’s own unmutated prediction rather than against experimental structures, which is a design choice that distinguishes this study from prior benchmarking work focused on predictive accuracy [23,43,90,91]. Our objective is not to assess whether AlphaFold 3 is correct, but whether its predictions change in proportion to the severity of sequence perturbation—a question that requires the unmutated prediction, not the experimental structure, as the appropriate reference. That being said, for the sake of completeness, we did also test the structural changes for AlphaFold and ESMFold with reference to the experimental PDB structure for each protein in the 200-protein dataset and found a similar trend of invariance. Refer to the Supplementary Materials for more details.

## Data Availability

The data used in the study are available on Zenodo with the DOI: 10.5281/zenodo.20752060. A full list of proteins used for benchmarking in the study is available in the Supplementary Materials.

## Supplementary Materials

Supplementary 1 Sections S1 to S3 Tables S1 to S9
