# Supplementary material for: Adversarial Sequence Mutations in AlphaFold and ESMFold Reveal Nonphysical Structural Invariance, Confidence Failures, and Concerns for Protein Design
Source: Comput Struct Biotechnol J. 2026 Jun 29;35(1):0142. doi: 10.34133/csbj.0142 (PMC13311257; doi:10.34133/csbj.0142)
Supplement: Supplementary 1 — Sections S1 to S3 Tables S1 to S9 [file csbj.0142.f1.pdf]

Supplementary Information: Adversarial Sequence Mutations in AlphaFold and ESMFold Reveal Nonphysical Structural Invariance, Confidence Failures, and Concerns for Protein Design

Jonathan Feldman[1,2,3], Maximilian Brogi[2,3], Jeffrey Skolnick[2,3*]

[1]College of Computing, Georgia Institute of Technology, Atlanta, Georgia, United States.
[2]Center for the Study of Systems Biology, Georgia Institute of Technology, Atlanta, Georgia, United States.
[3]School of Biological Sciences, Georgia Institute of Technology, Atlanta, Georgia, United States.

*Corresponding author(s). E-mail(s): skolnick@gatech.edu;
Contributing authors: jonathanfeldman@gatech.edu; maxi@gatech.edu;

S1 Fold-switching protein dataset

For the fifteen experimentally validated fold-switching proteins, mutations were applied with a modified position-weighting scheme that prioritized residues or regions identified in previous studies as inducing conformational transitions. Supplementary Table S7 specifies the mutation ranges and discrete positions for each protein. The weighting formula combined the center bias used for the main 200-protein dataset with an additional multiplier for experimentally validated regions:

$$w_i = \left[\epsilon + (1-\epsilon)\left(1 - \frac{|i-(L-1)/2|}{(L-1)/2}\right)\right] \times r_i$$

where $\epsilon = 0.1$ defines the edge penalty, and r_i is a range multiplier set to 1.5 for positions within specified mutation ranges and 1.0 otherwise. Discrete positions identified in the literature as critical for fold-switching were treated as mandatory

mutations and selected first before random sampling. This two-phase approach ensured that experimentally validated switch-inducing residues were always mutated while residues in broader functional regions received a 50% increased probability of selection, all while maintaining the center bias that preferentially targets core positions.

Model	Metric	Correlation	Coeff.	5%	10%	20%	40%	70%	Mean
AlphaFold 3	TM-score	Pearson	−0.66	0.77	0.71	0.63	0.49	0.32	0.58
		Spearman	−0.66	—	—	—	—	—	—
	lDDT	Pearson	−0.84	0.80	0.70	0.56	0.38	0.13	0.51
		Spearman	−0.84	—	—	—	—	—	—
	RMSD (Å)	Pearson	+0.46	6.80	7.29	11.35	12.43	15.64	10.70
		Spearman	+0.48	—	—	—	—	—	—
	Ranking score	Pearson	−0.57	0.77	0.73	0.71	0.62	0.51	0.67
		Spearman	−0.55	—	—	—	—	—	—
ESMFold	TM-score	Pearson	−0.67	0.74	0.69	0.54	0.34	0.27	0.52
		Spearman	−0.73	—	—	—	—	—	—
	lDDT	Pearson	−0.84	0.73	0.62	0.43	0.20	0.03	0.40
		Spearman	−0.88	—	—	—	—	—	—
	RMSD (Å)	Pearson	+0.49	5.58	8.20	10.76	15.90	16.35	11.36
		Spearman	+0.58	—	—	—	—	—	—

Table S1 Structural metrics for fold-switching proteins under point mutations for AlphaFold 3 and ESMFold. Correlation coefficients quantify the relationship between mutation level and metric degradation. Mean values are computed across fifteen experimentally validated fold-switching proteins from Porter et al. [1]. Ranking score is not applicable for ESMFold.

S2 SASA-Based Mutation Weighting: Validation and Results

S2.1 Motivation and Method

The primary mutation analyses in this study employed a sequence-center weighting scheme in which residues near the center of the primary sequence are assigned higher probabilities of selection, reflecting the assumption that central sequence positions are more likely to correspond to structurally important core residues. While computationally simple and often correct, this heuristic assumes core residues are defined not by burial in three-dimensional space but by proximity to the sequence midpoint. This correspondence breaks down for multi-domain proteins and those with atypical topologies.

To verify that the center-based heuristic does not materially bias our results, we repeated the mutation analyses using weights derived directly from per-residue solvent-accessible surface area (SASA), computed from the experimental structures. SASA was computed on the full biological assembly using the Shrake–Rupley

algorithm as implemented in BioPython [2], so that interface-buried residues in multimeric proteins are correctly identified as buried rather than exposed. For residues not resolved in the experimental structure, SASA values were assigned by linear interpolation between flanking resolved residues, with boundary extension for unresolved terminal segments. The resulting per-residue SASA values were converted to mutation-probability weights using the formula

$$w_i = \epsilon + (1 - \epsilon) \left(1 - \frac{\text{SASA}_i}{\max_j \text{SASA}_j}\right)$$

where $\epsilon = 0.1$ is the edge penalty, identical to the parameter used in the center-based scheme. This maps the most buried residue (SASA ≈ 0) to weight 1.0 and the most exposed residue to weight 0.1, preserving the same dynamic range as the original heuristic while grounding the weighting in structural depth rather than sequence position.

S2.2 Point Mutation Results Under SASA Weighting

Table S2 summarizes the aggregate point mutation results under SASA-based weighting across all 200 proteins. Mean TM-score, lDDT, RMSD, DockQ (multimers only), and AlphaFold 3 ranking score are reported at each mutation threshold, alongside Pearson and Spearman correlations with mutation percentage. The pattern of results mirrors that of the center-based analysis throughout: global fold is maintained on average up to the 40% mutation threshold (TM-score = 0.578), interfacial accuracy degrades rapidly for multimers, and confidence metrics decline gradually but remain in the high-confidence regime through 20% mutation. No meaningful differences were observed across the four protein categories (monomer-novel, monomer-similar, multimer-novel, multimer-similar).

**Metric**	**Pearson** r	**5%**	**10%**	**20%**	**40%**	**70%**
TM-score	−0.743	0.870	0.810	0.717	0.578	0.263
lDDT	−0.933	0.853	0.769	0.615	0.398	0.099
RMSD	+0.558	6.97	8.70	12.42	17.66	28.24
DockQ	−0.589	0.552	0.436	0.269	0.129	0.029
Ranking score	−0.558	0.742	0.700	0.622	0.516	0.330

Table S2 Point mutation results under SASA-based weighting. Mean metric values at each mutation threshold across all 200 proteins, with Pearson correlation between mutation percentage and each metric. DockQ values are reported for multimeric proteins only. RMSD is reported in Å.

S2.3 Deletion Mutation Results Under SASA Weighting

Table S3 presents the corresponding deletion mutation results. Again, the pattern is consistent with the center-based analysis: monomeric proteins maintain global fold accuracy through the 10% deletion threshold (TM-score = 0.561), multimeric interfacial accuracy degrades more rapidly, and confidence metrics show moderate sensitivity. lDDT again shows stronger correlation with deletion load than TM-score (Pearson $r = -0.805$ versus -0.498).

**Metric**	**Pearson r**	**1%**	**3%**	**5%**	**10%**
TM-score	−0.498	0.876	0.768	0.696	0.561
lDDT	−0.805	0.867	0.731	0.631	0.448
RMSD	+0.310	6.23	10.09	12.52	16.31
DockQ	−0.502	0.557	0.346	0.268	0.129
Ranking score	−0.405	0.738	0.655	0.592	0.472

Table S3 Values at each deletion threshold across all 200 proteins, with Pearson correlation between deletion percentage and each metric. DockQ values are reported for multimeric proteins only. RMSD is reported in Å.

S2.4 SASA-Analysis Findings

Despite per-residue weight disagreements between the two schemes, the SASA-based and center-based weighting schemes produce statistically indistinguishable results across all mutation levels, protein categories, and structural metrics. Both schemes preferentially target core positions relative to a uniform random baseline, and over 200 proteins and thousands of mutation events these individual differences average out. The center-based heuristic therefore serves as a valid and computationally efficient proxy for structural depth in the context of large-scale adversarial mutation analyses, and the results reported in the main text are robust to this methodological choice.

S3 Comparison Against Experimental Reference Structures

The primary mutation analyses in this study compare mutated predictions against the corresponding unmutated model prediction, which is the appropriate reference for measuring predictive invariance—that is, how much a model's output changes in response to sequence perturbation, independent of whether the original prediction was accurate. To confirm that the observed invariance is not an artifact of this self-referential design, we repeated the analysis using the experimental PDB structure as the reference for the 200-protein dataset and for the 100 monomeric proteins used in the ESMFold comparison. This approach addresses predictive accuracy rather than

invariance per se, and is therefore a distinct question from the one studied in the main text. Nevertheless, the pattern of structural preservation under mutation persists under this more stringent reference, reinforcing the conclusion that the findings are not an artifact of the experimental design.

For AlphaFold 3, the mean TM-score against the experimental structure remains above the 0.5 fold-preservation threshold through 40% point mutation (0.608) and through 10% deletion (0.592), closely mirroring the self-referential findings reported in the main text. ESMFold collapses below 0.5 between 20% and 40% point mutation—from 0.672 to 0.333—consistent with the earlier structural divergence documented in the main text relative to AlphaFold 3, which maintains a mean TM-score of 0.608 against the experimental reference at the same threshold. For deletion mutations, ESMFold's sensitivity is broadly comparable to AlphaFold 3's, with both models maintaining mean TM-scores above 0.5 through 10% deletion (0.592 for AlphaFold 3 and 0.583 for ESMFold). We note that ESMFold's mean RMSD at 70% point mutation (18.52 Å) is lower than at 40% (22.28 Å), which reflects a well-known artifact of superposition-based metrics at high structural divergence—when TM-score is very low, the rigid-body alignment underlying RMSD becomes unreliable. Full results are provided in Tables S4 and S5.

**Model**	**Metric**	**5%**	**10%**	**20%**	**40%**	**70%**
AlphaFold 3	TM-score	0.842	0.806	0.728	0.608	0.273
	lDDT	0.773	0.707	0.587	0.392	0.070
	RMSD (Å)	6.04	7.06	9.88	13.47	21.31
ESMFold	TM-score	0.783	0.767	0.672	0.333	0.258
	lDDT	0.645	0.591	0.445	0.126	0.016
	RMSD (Å)	6.79	7.10	10.10	22.28	18.52

Table S4 Point mutation results against experimental reference. Mean metric values at each mutation threshold for AlphaFold 3 across all 200 proteins and for ESMFold across 100 monomeric proteins. The non-monotonic RMSD at 70% reflects superposition failure at high structural divergence, where lDDT is the more appropriate metric.

Model	Metric	1%	3%	5%	10%
AlphaFold 3	TM-score	0.838	0.762	0.707	0.592
	lDDT	0.784	0.685	0.598	0.438
	RMSD (Å)	5.76	7.91	9.57	12.67
ESMFold	TM-score	0.798	0.749	0.691	0.583
	lDDT	0.666	0.573	0.483	0.333
	RMSD (Å)	6.06	7.16	8.57	11.72

Table S5 Deletion mutation results against experimental reference. Mean metric values at each deletion threshold for AlphaFold 3 across all 200 proteins and for ESMFold across 100 monomeric proteins.

Table S6 Complete structural metrics for AlphaFold 3 and ESMFold under adversarial point and deletion mutations. Mean metric values at each mutation threshold alongside Pearson correlation coefficients. AF3 results are reported across all 200 proteins; ESMFold results are restricted to 100 monomeric proteins. DockQ is reported for AF3 multimeric proteins only and is not applicable for ESMFold. All comparisons are against the respective model's unmutated prediction.

(A) AlphaFold 3 Point Mutations

**Metric**	**Pearson r**	**5%**	**10%**	**20%**	**40%**	**70%**
TM-score	−0.742	0.863	0.813	0.724	0.594	0.265
lDDT	−0.936	0.852	0.764	0.624	0.406	0.103
RMSD (Å)	+0.560	7.50	8.87	12.38	16.49	28.79
DockQ	−0.583	0.522	0.426	0.249	0.145	0.019
Ranking score	−0.558	0.730	0.694	0.620	0.521	0.318

(B) AlphaFold 3 Deletion Mutations

**Metric**	**Pearson r**	**1%**	**3%**	**5%**	**10%**
TM-score	−0.472	0.875	0.777	0.706	0.579
lDDT	−0.806	0.874	0.742	0.639	0.459
RMSD (Å)	+0.282	6.31	9.61	12.26	15.47
DockQ	−0.502	0.573	0.403	0.280	0.139
Ranking score	−0.412	0.755	0.676	0.602	0.484

(C) ESMFold Point Mutations

**Metric**	**Pearson r**	**5%**	**10%**	**20%**	**40%**	**70%**
TM-score	—	0.839	0.796	0.682	0.333	0.261
lDDT	—	0.772	0.685	0.495	0.138	0.021
RMSD (Å)	—	6.14	7.78	11.26	23.28	24.23

(D) ESMFold Deletion Mutations

**Metric**	**Pearson r**	**1%**	**3%**	**5%**	**10%**
TM-score	—	0.887	0.792	0.715	0.587
lDDT	—	0.832	0.679	0.561	0.371
RMSD (Å)	—	4.27	6.95	9.56	13.03

PDB (Chain A)	Predicted in [1]	Manual Search	Citation(s)
2KXO	1–89	2–30, 24, 25	[3, 4]
2LSH	29–115	—	[5]
4OV8	247–318	128–147, 298–313	[6]
2MZ7	267–312	275–280, 306–311	[7]
4PMK	27–62	—	[8]
2N4O	16–69	—	[9]
2KTM	167–201	182–217	[10]
2LE3	N/A	19–30, 24	[11]
2X9C	N/A	69–70, 71–76	[12]
3J9E	2–71	1–68, 69–354	[13]
5SUZ	474–509, 415–509	436; 442–447, 499, 460	[14]
4HLS	146–222	170, 174	[15]
1S5P	48–107, 98–189, 208–274	—	[16]
3TKA	236–313	—	[17]
3GAX	48–120	43–59	[18]

Table S7 Manually identified residues or regions associated with fold switching proteins as collated by [1]. Entries marked with dashes (—) indicate that no precise fold-switching region was found.

From	To	From	To	From	To	From	To
S	W	N	A	K	G	F	T
T	F	Q	G	R	A	M	Q
C	W	Y	G	W	S	L	N
D	W	H	A	A	Q	I	N
E	F	G	K	V	E	P	R

Table S8 Point mutation transformation pairs used for maximal disruption to protein structures. Standard abbreviations are used for each of the 20 canonical amino acids.

	Metric	Pearson r	p-value	Spearman ρ	p-value
Monomers	Top 1 mean	0.320	1.5×10^{-3}	0.352	4.4×10^{-4}
	Top 5 mean	0.324	1.3×10^{-3}	0.333	9.2×10^{-4}
	Top 10 mean	0.296	3.5×10^{-3}	0.308	2.3×10^{-3}
	Top 20 mean	0.253	0.013	0.269	8.0×10^{-3}
	Top 100 mean	0.259	0.011	0.317	1.6×10^{-3}
	Drop (1→100)	0.037	0.722	−0.038	0.715
Multimers	Top 1 mean	0.268	7.7×10^{-3}	0.296	3.1×10^{-3}
	Top 5 mean	0.278	5.7×10^{-3}	0.360	2.7×10^{-4}
	Top 10 mean	0.294	3.3×10^{-3}	0.395	5.8×10^{-5}
	Top 20 mean	0.333	8.1×10^{-4}	0.430	9.9×10^{-6}
	Top 100 mean	0.382	1.1×10^{-4}	0.457	2.3×10^{-6}
	Drop (1→100)	−0.168	0.099	−0.153	0.133

Table S9 Correlation between AlphaFold 3 confidence and template neighborhood statistics for monomers ($n = 96$) and multimers ($n = 98$). Top N refers to the mean query-normalized TM-score of the N best pre-cutoff structural matches. Monomer and multimer confidence is measured by AlphaFold 3 ranking score.

References

[1] Porter, L.L., Looger, L.L.: Extant fold-switching proteins are widespread. Proceedings of the National Academy of Sciences **115**(23), 5968–5973 (2018) https://doi.org/10.1073/pnas.1800168115 . Publisher: Proceedings of the National Academy of Sciences. Accessed 2025-12-02

[2] Cock, P.J.A., Antao, T., Chang, J.T., Chapman, B.A., Cox, C.J., Dalke, A., Friedberg, I., Hamelryck, T., Kauff, F., Wilczynski, B., Hoon, M.J.L.: Biopython: freely available python tools for computational molecular biology and bioinformatics. Bioinformatics **25**(11), 1422–1423 (2009) https://doi.org/10.1093/bioinformatics/btp163 https://academic.oup.com/bioinformatics/article-pdf/25/11/1422/48989335/bioinformatics$_25_11_1422$*.pdf*

[3] Park, K.-T., Wu, W., Battaile, K.P., Lovell, S., Holyoak, T., Lutkenhaus, J.: The min oscillator uses mind-dependent conformational changes in mine to spatially regulate cytokinesis. Cell **146**(3), 396–407 (2011)

[4] Ayed, S.H., Cloutier, A.D., McLeod, L.J., Foo, A.C., Damry, A.M., Goto, N.K.: Dissecting the role of conformational change and membrane binding by the bacterial cell division regulator mine in the stimulation of mind atpase activity. Journal of Biological Chemistry **292**(50), 20732–20743 (2017)

[5] Morris, V.K., Kwan, A.H., Sunde, M.: Analysis of the structure and conformational states of dewa gives insight into the assembly of the fungal hydrophobins. Journal of molecular biology **425**(2), 244–256 (2013)

[6] Lukoyanova, N., Kondos, S.C., Farabella, I., Law, R.H., Reboul, C.F., Caradoc-Davies, T.T., Spicer, B.A., Kleifeld, O., Traore, D.A., Ekkel, S.M., *et al.*: Conformational changes during pore formation by the perforin-related protein pleurotolysin. PLoS biology **13**(2), 1002049 (2015)

[7] Kadavath, H., Jaremko, M., Jaremko, Ł., Biernat, J., Mandelkow, E., Zweckstetter, M.: Folding of the tau protein on microtubules. Angewandte Chemie International Edition **54**(35), 10347–10351 (2015)

[8] Hamiaux, C., Maddumage, R., Middleditch, M.J., Prakash, R., Brummell, D.A., Baker, E.N., Atkinson, R.G.: Crystal structure of kiwellin, a major cell-wall protein from kiwifruit. Journal of structural biology **187**(3), 276–281 (2014)

[9] Pham, C.L., Rey, A., Lo, V., Soulès, M., Ren, Q., Meisl, G., Knowles, T.P., Kwan, A.H., Sunde, M.: Self-assembly of mpg1, a hydrophobin protein from the rice blast fungus that forms functional amyloid coatings, occurs by a surface-driven mechanism. Scientific reports **6**(1), 25288 (2016)

[10] Adrover, M., Pauwels, K., Prigent, S., Chiara, C., Xu, Z., Chapuis, C., Pastore, A., Rezaei, H.: Prion fibrillization is mediated by a native structural element that

comprises helices h2 and h3. Journal of Biological Chemistry **285**(27), 21004–21012 (2010)

[11] Rao, J.N., Warren, G.Z., Estolt-Povedano, S., Zammit, V.A., Ulmer, T.S.: An environment-dependent structural switch underlies the regulation of carnitine palmitoyltransferase 1a*. Journal of Biological Chemistry **286**(49), 42545–42554 (2011)

[12] Poyraz, Ö., Schmidt, H., Seidel, K., Delissen, F., Ader, C., Tenenboim, H., Goosmann, C., Laube, B., Thünemann, A.F., Zychlinsky, A., *et al.*: Protein refolding is required for assembly of the type three secretion needle. Nature structural & molecular biology **17**(7), 788–792 (2010)

[13] Zhang, X., Patel, A., Celma, C.C., Yu, X., Roy, P., Zhou, Z.H.: Atomic model of a nonenveloped virus reveals ph sensors for a coordinated process of cell entry. Nature structural & molecular biology **23**(1), 74–80 (2016)

[14] Gammons, M.V., Renko, M., Johnson, C.M., Rutherford, T.J., Bienz, M.: Wnt signalosome assembly by dep domain swapping of dishevelled. Molecular cell **64**(1), 92–104 (2016)

[15] Sweeting, B., Brown, E., Khan, M.Q., Chakrabartty, A., Pai, E.F.: N-terminal helix-cap in α-helix 2 modulates β-state misfolding in rabbit and hamster prion proteins. PloS one **8**(5), 63047 (2013)

[16] Zhao, K., Chai, X., Marmorstein, R.: Structure and substrate binding properties of cobb, a sir2 homolog protein deacetylase from escherichia coli. Journal of molecular biology **337**(3), 731–741 (2004)

[17] Wei, Y., Zhang, H., Gao, Z.-Q., Wang, W.-J., Shtykova, E.V., Xu, J.-H., Liu, Q.-S., Dong, Y.-H.: Crystal and solution structures of methyltransferase rsmh provide basis for methylation of c1402 in 16s rrna. Journal of structural biology **179**(1), 29–40 (2012)

[18] Szymańska, A., Jankowska, E., Orlikowska, M., Behrendt, I., Czaplewska, P., Rodziewicz-Motowidło, S.: Influence of point mutations on the stability, dimerization, and oligomerization of human cystatin c and its l68q variant. Frontiers in Molecular Neuroscience **5**, 82 (2012)
